# Supplementary material for: Role of pericytes in blood–brain barrier preservation during ischemia through tunneling nanotubes
Source: Cell Death Dis. 2022 Jul 5;13(7):582. doi: 10.1038/s41419-022-05025-y (PMC9256725; doi:10.1038/s41419-022-05025-y)
Supplement: Supplementary file 21 — Supplementary Movies 8,9 [file 41419_2022_5025_MOESM21_ESM.pptx]

## Slide 1
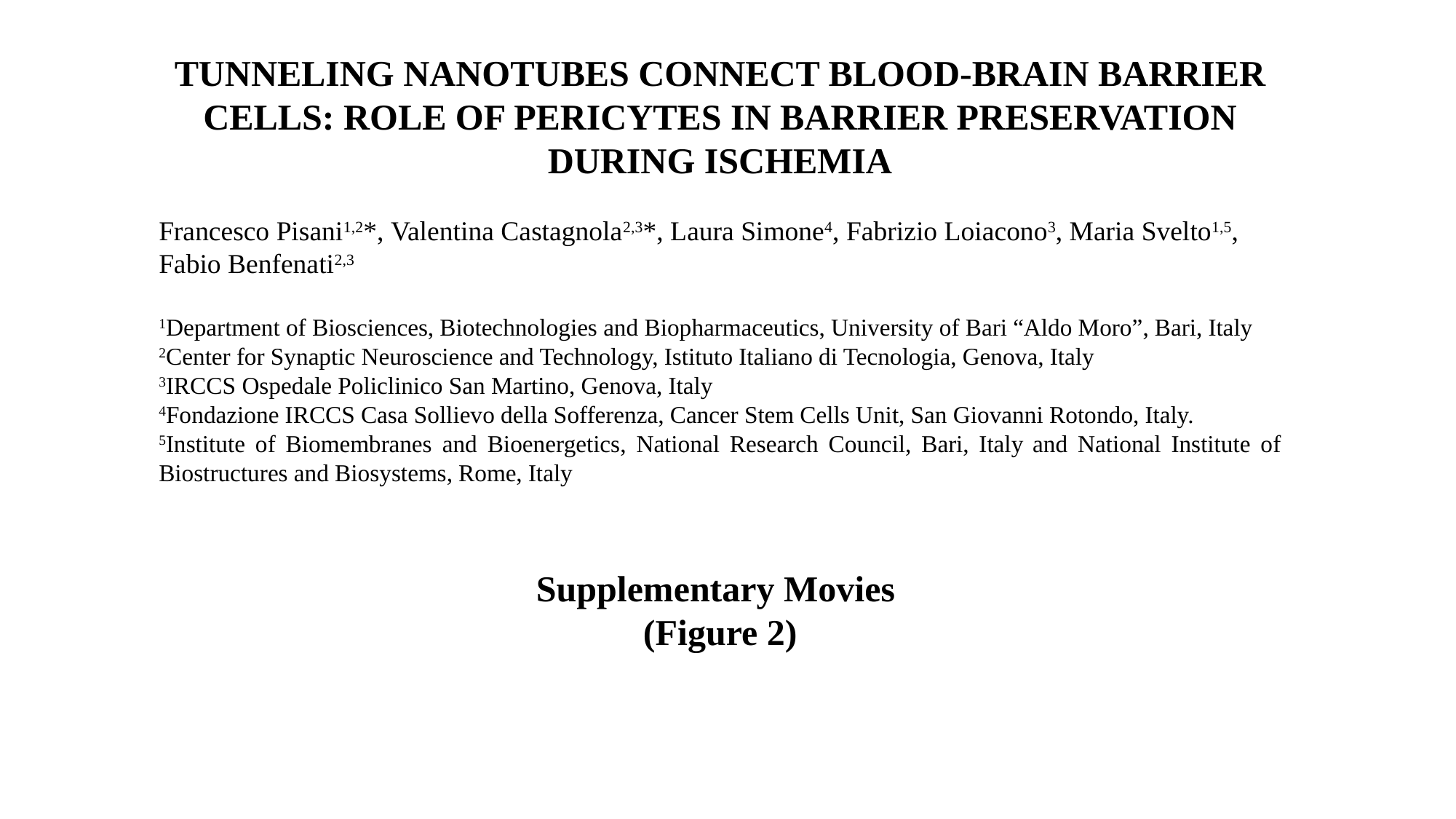

TUNNELING NANOTUBES CONNECT BLOOD-BRAIN BARRIER CELLS: ROLE OF PERICYTES IN BARRIER PRESERVATION DURING ISCHEMIA
Francesco Pisani1,2*, Valentina Castagnola2,3*, Laura Simone4, Fabrizio Loiacono3, Maria Svelto1,5, Fabio Benfenati2,3
1Department of Biosciences, Biotechnologies and Biopharmaceutics, University of Bari “Aldo Moro”, Bari, Italy
2Center for Synaptic Neuroscience and Technology, Istituto Italiano di Tecnologia, Genova, Italy
3IRCCS Ospedale Policlinico San Martino, Genova, Italy
4Fondazione IRCCS Casa Sollievo della Sofferenza, Cancer Stem Cells Unit, San Giovanni Rotondo, Italy.
5Institute of Biomembranes and Bioenergetics, National Research Council, Bari, Italy and National Institute of Biostructures and Biosystems, Rome, Italy
Supplementary Movies
(Figure 2)

## Slide 2
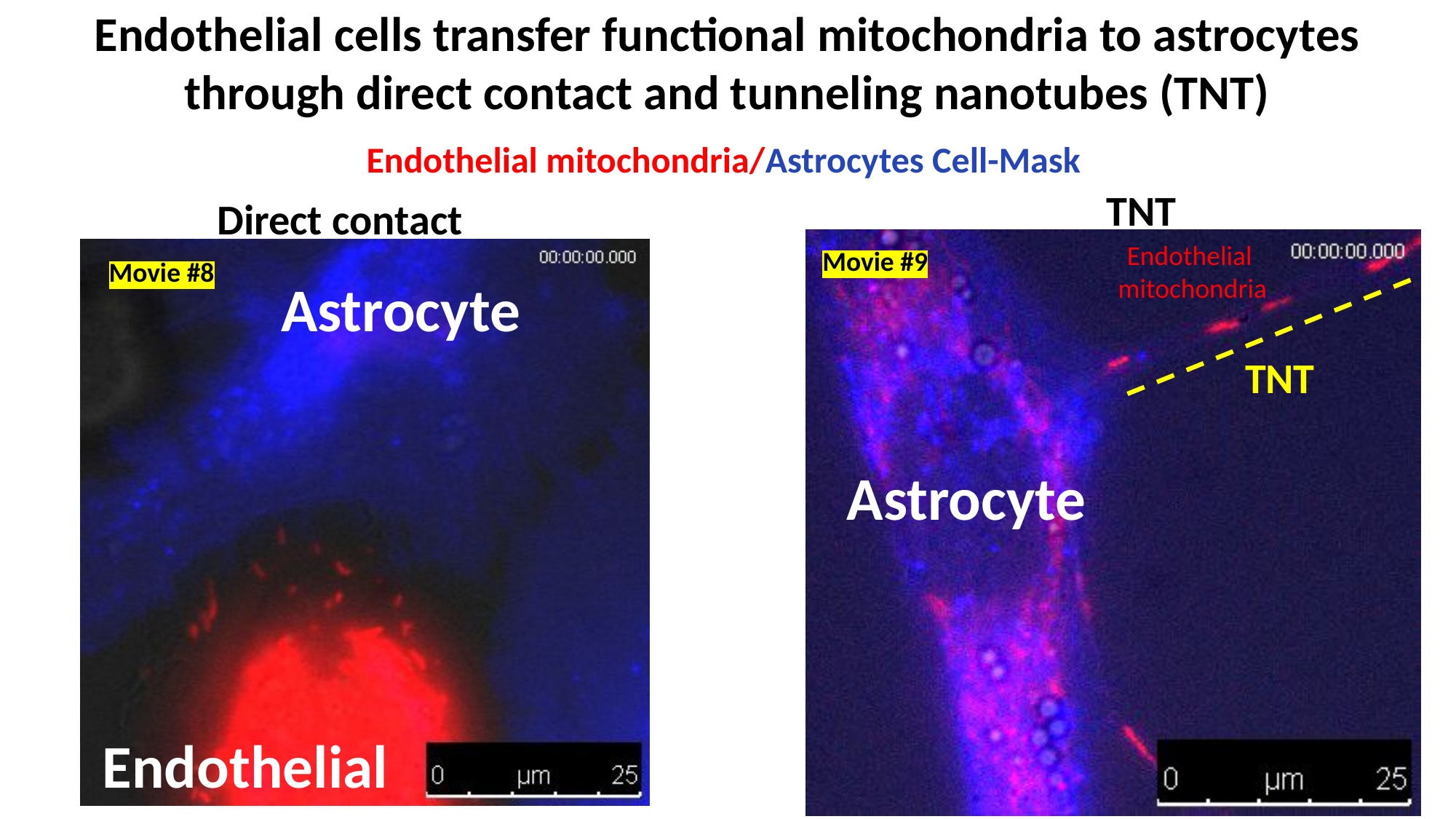

Endothelial cells transfer functional mitochondria to astrocytes through direct contact and tunneling nanotubes (TNT)
Endothelial mitochondria/Astrocytes Cell-Mask
TNT
Direct contact
Endothelial
mitochondria
Movie #9
Movie #8
Astrocyte
TNT
Astrocyte
Endothelial
