# Supplementary material for: Role of pericytes in blood–brain barrier preservation during ischemia through tunneling nanotubes
Source: Cell Death Dis. 2022 Jul 5;13(7):582. doi: 10.1038/s41419-022-05025-y (PMC9256725; doi:10.1038/s41419-022-05025-y)
Supplement: Supplementary file 23 — Supplementary Movies 14-16 [file 41419_2022_5025_MOESM23_ESM.pptx]

## Slide 1
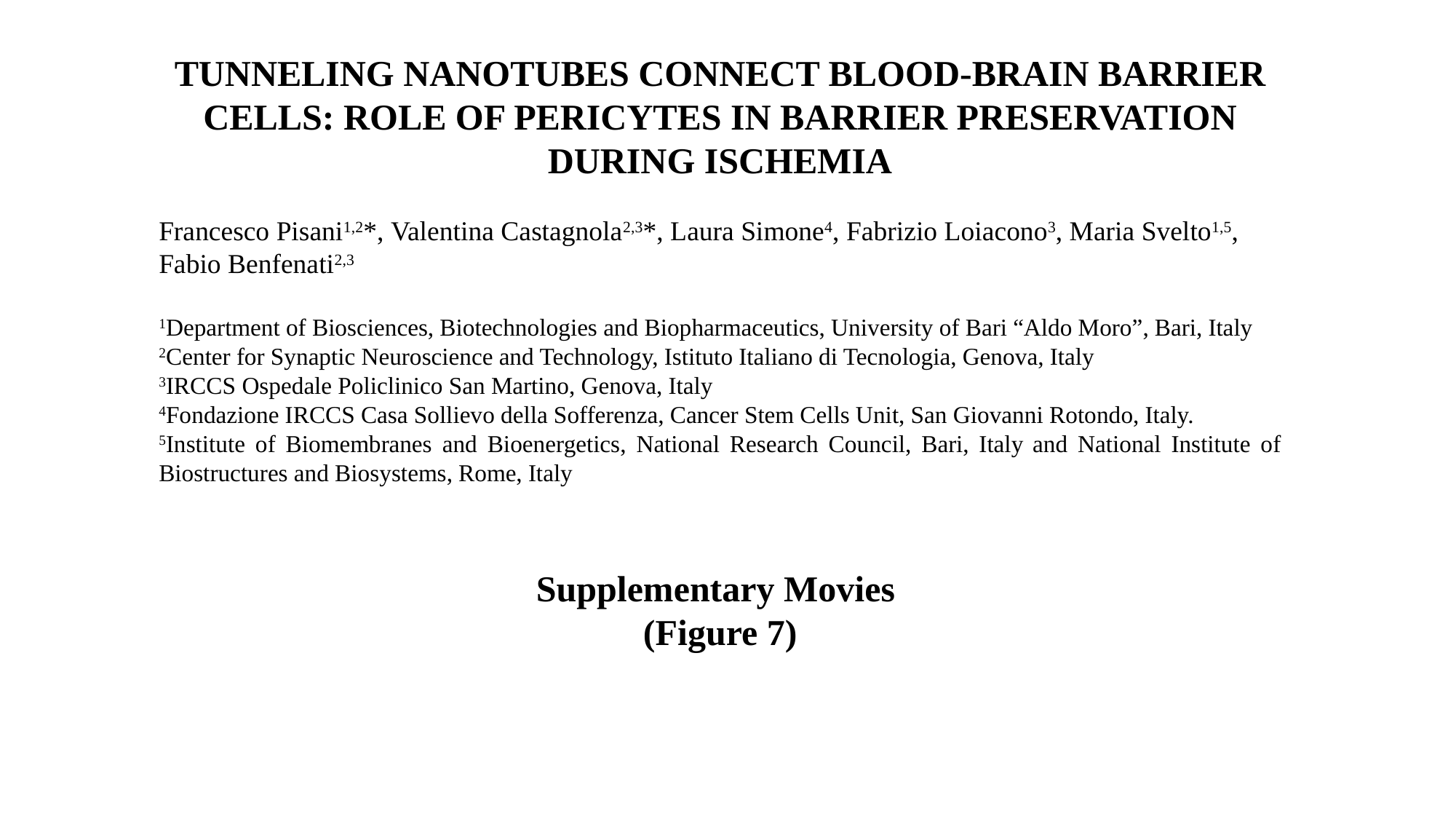

TUNNELING NANOTUBES CONNECT BLOOD-BRAIN BARRIER CELLS: ROLE OF PERICYTES IN BARRIER PRESERVATION DURING ISCHEMIA
Francesco Pisani1,2*, Valentina Castagnola2,3*, Laura Simone4, Fabrizio Loiacono3, Maria Svelto1,5, Fabio Benfenati2,3
1Department of Biosciences, Biotechnologies and Biopharmaceutics, University of Bari “Aldo Moro”, Bari, Italy
2Center for Synaptic Neuroscience and Technology, Istituto Italiano di Tecnologia, Genova, Italy
3IRCCS Ospedale Policlinico San Martino, Genova, Italy
4Fondazione IRCCS Casa Sollievo della Sofferenza, Cancer Stem Cells Unit, San Giovanni Rotondo, Italy.
5Institute of Biomembranes and Bioenergetics, National Research Council, Bari, Italy and National Institute of Biostructures and Biosystems, Rome, Italy
Supplementary Movies
(Figure 7)

## Slide 2
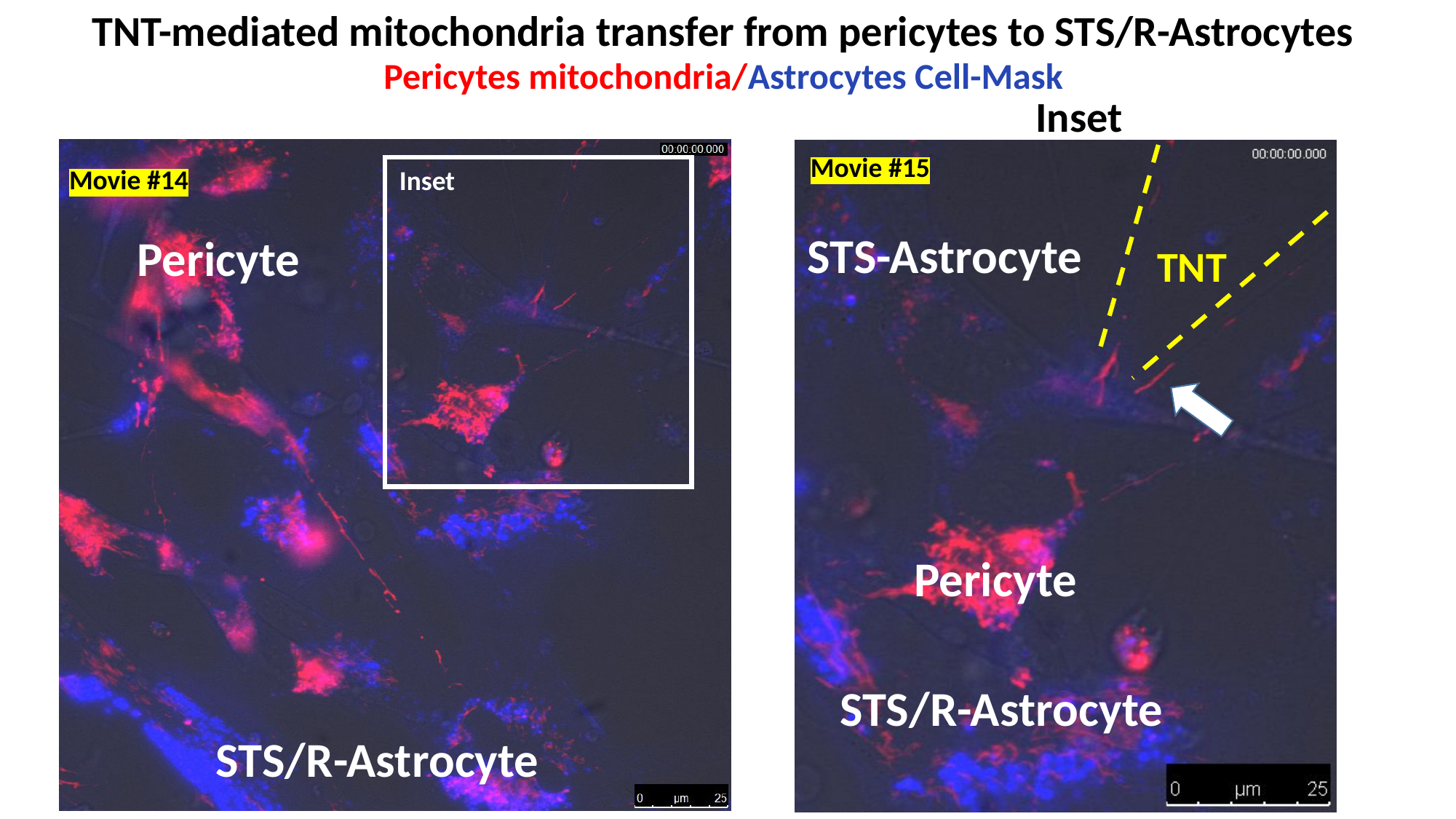

TNT-mediated mitochondria transfer from pericytes to STS/R-Astrocytes
Pericytes mitochondria/Astrocytes Cell-Mask
Inset
Movie #15
Movie #14
Inset
STS-Astrocyte
Pericyte
TNT
Pericyte
STS/R-Astrocyte
STS/R-Astrocyte

## Slide 3
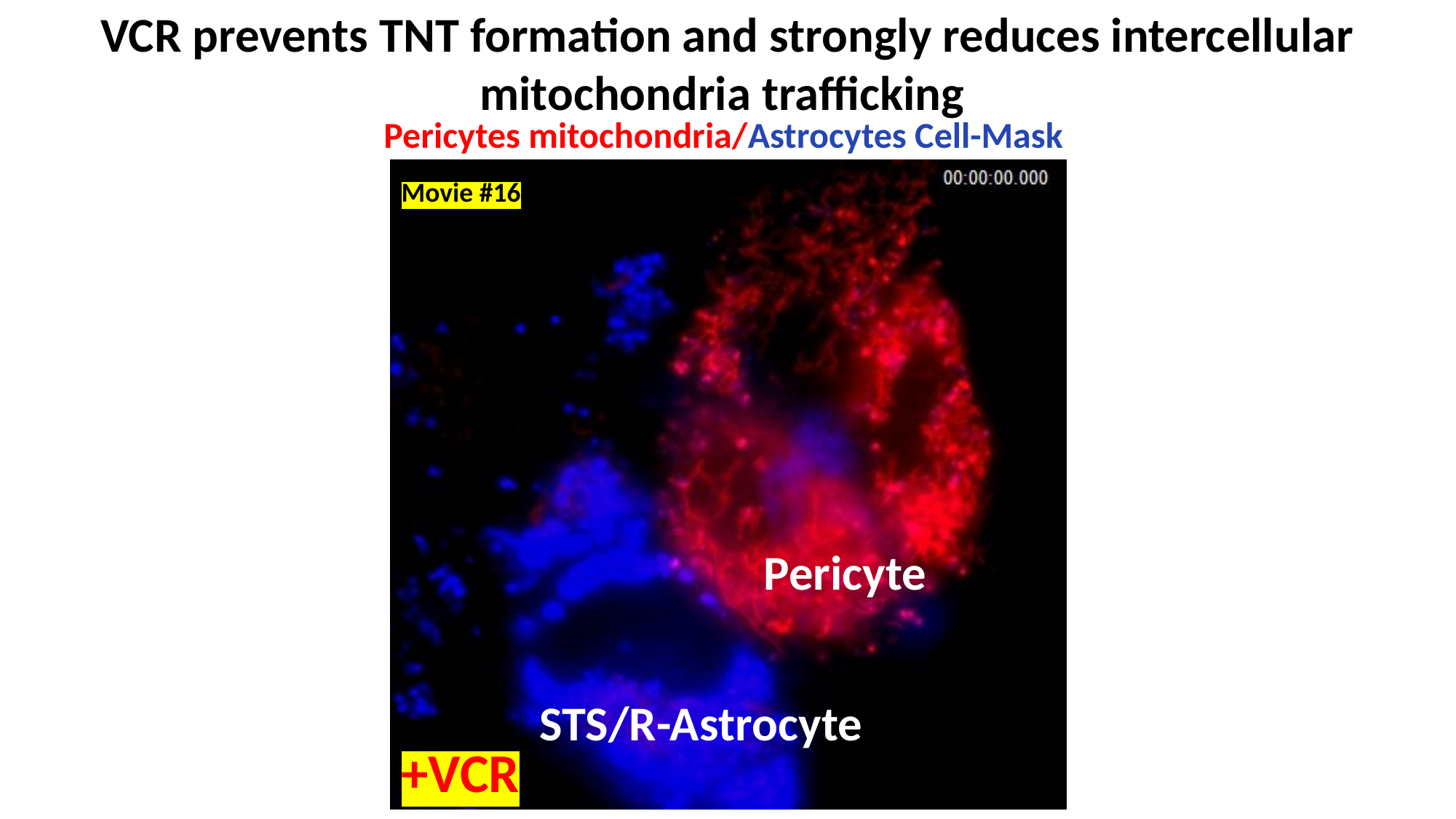

VCR prevents TNT formation and strongly reduces intercellular mitochondria trafficking
Pericytes mitochondria/Astrocytes Cell-Mask
Movie #16
Pericyte
STS/R-Astrocyte
+VCR
